# Transcriptome in Liver of Periparturient Dairy Cows Differs between Supplementation of Rumen-Protected Niacin and Rumen-Protected Nicotinamide

**DOI:** 10.3390/metabo14030150

**Published:** 2024-03-01

**Authors:** Yuanjie Zhang, Rongrong Li, Xue’er Du, Zhijie Cui, Xingwei Jiang, Lamei Wang, Junhu Yao, Shimin Liu, Jianguo Wang, Chuanjiang Cai, Yangchun Cao

**Affiliations:** 1 College of Animal Science and Technology, Northwest A&F University, Xianyang 712100, China; 2 UWA Institute of Agriculture, The University of Western Australia, Crawley, WA 6009, Australia; 3 College of Veterinary Medicine, Northwest A&F University, Xianyang 712100, China

**Keywords:** rumen-protected niacin, rumen-protected nicotinamide, transcriptomic, lipid metabolism, liver, periparturient dairy cows

## Abstract

To investigate the difference between rumen-protected niacin (RPN) and rumen-protected nicotinamide (RPM) in the transcriptome of genes relating to the lipid metabolism of the liver of periparturient dairy cows, 10 healthy Chinese Holstein cows were randomly divided into two groups and fed diets supplemented with 18.4 g/d RPN or 18.7 g/d RPM, respectively. The experiment lasted from 14 days before to 21 days after parturition. Liver biopsies were taken 21 days postpartum for transcriptomic sequencing. In addition, human LO2 cells were cultured in a medium containing 1.6 mmol/L of non-esterified fatty acids and 1 mmol/L niacin (NA) or 2 mmol/L nicotinamide (NAM) to verify the expression of the 10 genes selected from the transcriptomic analysis of the liver biopsies. The expression of a total of 9837 genes was detected in the liver biopsies, among which 1210 differentially expressed genes (DEGs) were identified, with 579 upregulated and 631 downregulated genes. These DEGs were associated mainly with lipid metabolism, oxidative stress, and some inflammatory pathways. Gene ontology (GO) enrichment analysis showed that 355 DEGs were enriched in 38 GO terms. The differences in the expression of these DEGs between RPN and RPM were predominantly related to the processes of steroid catabolism, steroid hydroxylase, monooxygenase activity, oxidoreductase activity, hemoglobin binding, and ferric iron binding, which are involved mainly in lipid anabolism and redox processes. The expressions of *FADS2*, *SLC27A6*, *ARHGAP24*, and *THRSP* in LO2 cells were significantly higher (*p* < 0.05) while the expressions of *BCO2, MARS1, GARS1, S100A12*, *AGMO*, and *OSBPL11* were significantly lower (*p* < 0.05) on the NA treatment compared to the NAM treatment, indicating that NA played a role in liver metabolism by directly regulating fatty acid anabolism and transport, inflammatory factor expression, and oxidative stress; and NAM functioned more as a precursor of nicotinamide adenine dinucleotide (NAD, coenzyme I) and nicotinamide adenine dinucleotide phosphate (NADP, coenzyme II) to participate indirectly in biological processes such as ether lipid metabolism, cholesterol metabolism, energy metabolism, and other processes.

## 1. Introduction

The transition period in dairy cows (21 days, respectively, before and after calving) is the most critical stage of the lactation cycle. Approximately 75% of diseases in cows occurs during this period, and severe diseases can lead to the culling or even death of cattle, resulting in significant economic losses [1]. Energy deficiency is the main cause of diseases in periparturient cows [2]. As a result, a large amount of body fat is mobilized to compensate for the energy deficit [3]. An increase in fat mobilization in the body results in a significant increase in plasma non-esterified fatty acids (NEFAs), beta-hydroxybutyrate (BHBA), and hepatic triglycerides (TAGs). High concentrations of NEFAs are highly associated with the incidence of ketosis and fatty liver disease in lactating cows [4], as well as oxidative stress, protein metabolism disorder, and inflammation [5], which results in a reduction in lactating performance, fertility and immunity, and economic income. Niacin (NA) and its amide derivative nicotinamide (NAM) are two forms of water-soluble vitamin B3, which is also known as vitamin PP, an indispensable nutrient in dairy cows. A sharp decline in feed intake in perinatal cows is accompanied with reduction in dietary intake of niacin, therefore, additional dietary niacin must be supplied to meet the cows’ demand for this vitamin [6]. Morey reported that addition of 24 g/d of RPN to the basal diet for periparturient dairy cows reduced postpartum plasma NEFA concentrations [7]. 

The major role of NA is anti-lipolytic [8], which can be used to alleviate hepatic lipid deposition by increasing hepatic lipid oxidation and decreasing de novo fatty acid synthesis. The role is mainly mediated through the niacin receptor, the G-protein–coupled receptor (GPCR) GPR109A, as well as by non-competitively inhibiting the activity of key rate-limiting enzymes in hepatic triglyceride synthesis [9]. In addition to its role in lipid metabolism, NA also improves dyslipidemia and reduces inflammation in adipose tissues by affecting key genes involved in gluconeogenesis, which in turn modulate the expression of genes associated with glycolysis, the pentose phosphate pathway, lipid and cholesterol synthesis, lipid transport, and very-low-density lipoprotein (VLDL)/LDL assembly [6,7,8,10,11,12]. Excessive accumulation of liver fat leads to elevated levels of reactive oxygen species(ROS) and exacerbates inflammation, while the addition of NA into cultured hepatocytes alleviated lipid accumulation, inhibited ROS production, and reduced the levels of pro-inflammatory factors [13].Unlike NA, NAM can reduce blood fatty acid content by promoting lipid synthesis, and perinatal supplementation of NAM facilitates the transformation of the rumen fermentation mode to the propionic acid type, which not only improves the efficiency of liver energy metabolism, but also promotes lipid metabolism [14]. NAM treatment increases proteins related to mitochondrial function that are involved in oxidative phosphorylation, fatty acid oxidation, and the tricarboxylic acid (TCA) cycle, thereby enhancing cellular antioxidant capacity [9,15]. Therefore, the addition of high doses of NA and NAM can indeed significantly improve the nutritional and health status of dairy cows, such as promoting rumen digestion, improving colostrum quality, increasing milk production, enhancing antioxidant and immune capacity [6,16,17,18,19], and reducing plasma NEFA content and insulin resistance [13,19].

Furthermore, NA and NAM are components of nicotinamide adenine dinucleotide (NAD, coenzyme I) and nicotinamide adenine dinucleotide phosphate (NADP, coenzyme II). These coenzymes are involved in the regulation of energy metabolism, glycolipid metabolism, inflammation, and oxidative stress in the organism [20]. The metabolism of NA and NAM produces NAD^+^, which serves as a necessary catalyst in energy production pathways, including glycolysis, the TCA cycle, fatty acid oxidation, and oxidative metabolism.

To investigate the transcriptomic differences between dietary addition of RPN and RPM on hepatic lipid metabolism in periparturient dairy cows, we performed a biopsy of cow livers to identify differentially expressed genes (DEGs) and screen for differential metabolic pathways. Then, we validated some DEGs in an in vitro model constructed from NEFA-treated cells to further understand the different mechanisms of the nutritional action of NA and NAM at the molecular level. Currently, bovine hepatocytes are not easily available, so we used human hepatocyte line 2 (LO2) cells in this experiment to build the model and treated the cells with NA or NAM to explore the molecular mechanisms.

## 2. Materials and Methods

### 2.1. Animals and Sample Collection

All experimental protocols and interventions in this study were approved by the Ethics Committee of the Northwest A&F University and complied with the Animal Protection Act and the Guidelines for the Care and Use of Laboratory Animals.

Ten healthy Chinese Holstein pregnant cows does with similar parity, weight and expected calving date were selected and randomly divided into 2 groups (5 pregnant cows per group): RPN (RPN 18.4 g/d, purity 65%, rumen bypass rate 80.3%, active ingredient 9.6 g/d) and RPM (RPM 18.7 g/d, purity 60%, rumen bypass rate 85.6%, active ingredient 9.6 g/d) from King Techina, Hangzhou, China. The cows were fed for a period of 35 days, including 14 days before calving and 21 days after calving. During the experimental period, the cows gave birth to healthy calves according to the pre-calving schedule.

All cows were housed in an individual pen and fed twice a day at 07:00 and 16:00 h with a total mixed diet ration (TMR). Pregnant cows were fed a prepartum diet, and after calving they were fed a postpartum diet. The ingredients and chemical compositions of the diet are shown in Table 1. The cows were allowed to eat and drink ad libitum. Liver biopsy was performed by hepatic needle aspiration on day 21 postpartum (collected at 13:00 each day) as previously described by Shen et al. [21]. Then, the liver biopsies were frozen immediately after collection in liquid nitrogen and stored at −80 °C.

### 2.2. Culture of Human Hepatocyte Line LO2

LO2 cells were initially cultured at 37 °C for 24 h at 5% CO_2_ in Roswell Park Memorial Institute (RPMI) 1640 medium (HyClone, Logan, UT, USA) containing 10% fetal bovine serum (Gibco BRL, Grand Island, NY, USA). LO2 cells were placed in a 1.6 mmol/L NEFA-deficient RPMI 1640 medium (without NA and NAM) to establish a fatty liver cell model, and then 1 mmol/L NA or 2 mmol/L NAM was added to the cells. The stock NEFA solution contained 3.19 mM palmitic acid, 0.53 mM palmitoleic acid, 1.44 mM stearic acid, 0.49 mM linoleic acid, and 4.35 mM oleic acid, and the final concentration of the stock solution was 53.4 mM. All reagents were purchased from Sigma-Aldrich.

### 2.3. RNA Isolation and Quantitative Real-Time Polymerase Chain Reaction

Total RNA from LO2 cells was isolated with Trizol (Invitrogen). The Reverse Transcription-Polymerase Chain Reaction (RT-PCR) reaction conditions were carried out according to the instructions provided by Vazyme. 1–2 µg of total RNA was treated with DNAse (Invitrogen) and then reverse transcribed to cDNA using Moloney Murine Leukemia Virus Reverse Transcriptase (M-MLV) reverse transcriptase (Vazyme, Nanjing, China). Quantitative Real-Time PCR (RT-PCR) was performed using SYBR^®^ Select Master Mix (vazyme, Nanjing, China) and BIO-RAD^®^ (Funglyn Biotech Inc., Richmond Hill, ON, Canada) CFX Opus 96 Real-Time PCR System. The relative quantitative gene expression levels were determined using the 2^−ΔΔCt^ method and normalized by *GAPDH* (Table 2). 

### 2.4. RNA Isolation and cDNA Library Construction

Total RNA in liver biopsies was extracted, and the RNA concentration and purity were determined using a Nanodrop2000 (Thermo Fisher Scientific, Waltham, MA, USA). Agarose gel- electrophoresis was used to detect the integrity of RNA. An Agilent 2100 Bioanalyzer was used to measure the RNA value. The enriched mRNA was segmented, then reverse synthesized to cDNA, which completed the construction of the library to be sequenced. The following conditions were set up for the creation of a single library to be sequenced: the total amount of RNA ≤ 1 µg, the concentration ≥ 35 ng/µL, OD260/280 ≥ 1.8, OD260/230 ≥ 1.0. After passing the quality assessment, the library was sequenced on the IlluminaNovaseq6000 platform, and the database was established by Shanghai Meiji Biomedical Technology Co., Shanghai, China.

### 2.5. Analysis of Differential Expression of Genes

The software DESeq2 version1.24.0 in R [22] based on negative binomial distribution was used to analyze the DEGs. We used the NA-treated group as a control for comparison between the two groups. According to the *p*-value and |log_2_ FC| (fold change) as screening thresholds, log_2_ FC > 0 indicates gene expression as upregulated and log_2_ FC < 0 indicates gene expression as downregulated.

### 2.6. Analysis of DEG Enrichment

Goatools software version 0.6.5 in python [23] was used for GO (gene ontology) enrichment analysis of genes/transcripts, and R script was used for KEGG (Kyoto encyclopedia of genes and genomes) pathway enrichment analysis of genes/transcripts. Fisher’s exact test and the Benjamini–Hochberg method was used to correct the *p*-value. Significance of GO enrichment and KEGG enrichment was considered as a *p*-value < 0.05. 

### 2.7. Statistical Analysis

MetaboAnalyst 5.0 (Wishart Research Group, Edmonton, AB, Canada) software was used to perform orthogonal partial least squares-discriminant analysis (OPLS-DA) on the samples from the RPN and RPM treatments. A volcano plot was used to analyze DEGs and their changes in each treatment, and a cluster heatmap was used to analyze the gene expression patterns in each sample. Statistical analysis was performed using IBM SPSS Statistics 26.0 (Armonk, NY, USA). Data are represented as means ± SD and treated with normal distribution test. Unpaired Student’s *t*-test was used to determine the significance between the means of RPN and RPM treatments. * indicates *p* < 0.05, ** indicates *p* < 0.01, *** indicates *p* < 0.001.

## 3. Results

### 3.1. Sequencing Data Quality Control

Transcriptome sequencing data showed better results for subsequent analysis (Supplemental Table S1).

### 3.2. Analysis of DEGs

**Figure 1:**
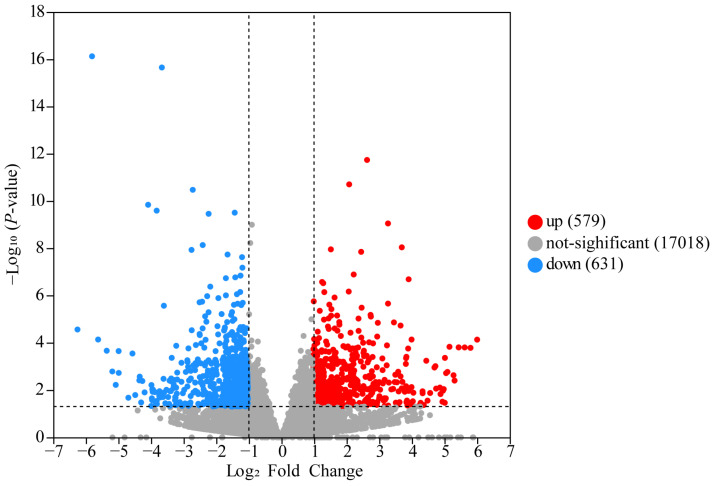
Volcano plot of DEGs. The corrected *p*-value is *p*-adjust. Each dot represents a gene in which red represents significant upregulation of gene expression and blue represents significant downregulation of gene expression. The closer the point is to the two sides and the upper side, the more significant the difference is.

**Figure 2:**
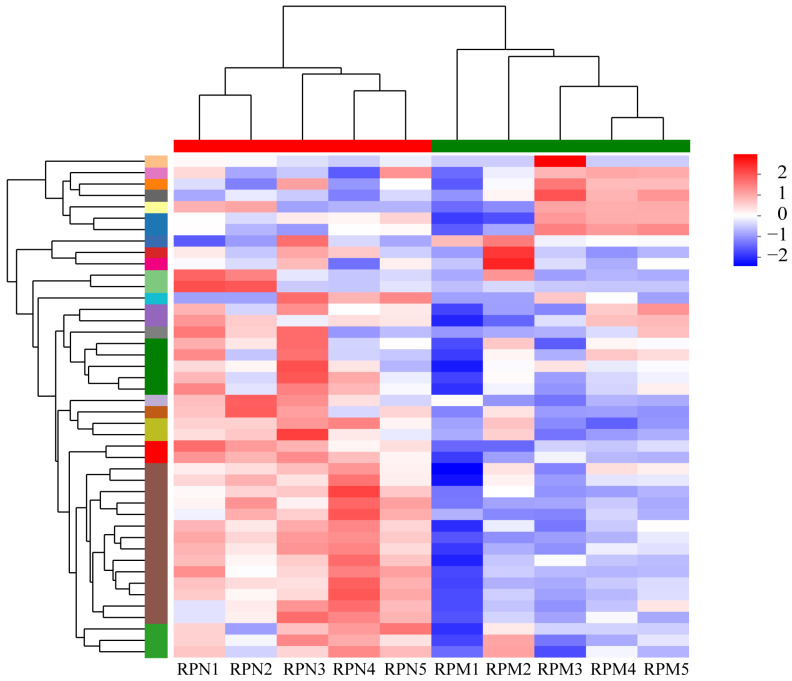
Cluster heatmap of DEGs in different samples. Different colors represent the expression value of the gene after standardization in each sample. On the left side is the tree diagram of the gene cluster and the module diagram of the sub cluster. The closer the two gene branches are, the closer their expression amounts are. The upper side in this graph is the tree diagram of the sample cluster. The closer the two samples branch, the closer the expression patterns of all genes in the two samples are. RPN = rumen-protected niacin group; RPM = rumen-protected nicotinamide group.

**Figure 3:**
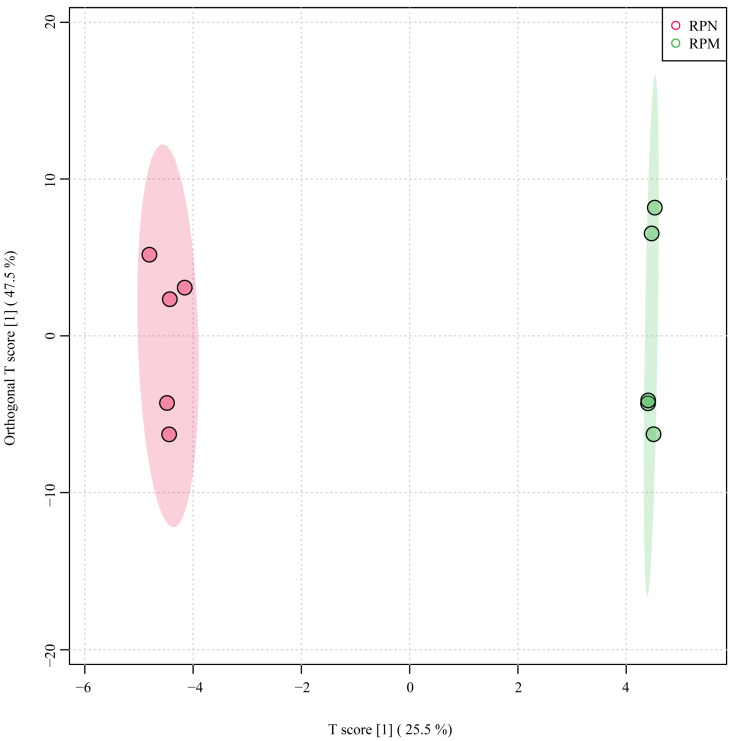
OPLS-DA score plot of different samples. RPN = rumen-protected niacin group; RPM = rumen-protected nicotinamide group.

The transcriptome gene expression results of the RPN and RPM groups showed that 1261 genes were specifically expressed in the RPN treatment, and 358 genes were specifically expressed in the RPM treatment (Supplementary Figure S1). To characterize the differences in transcriptional profiles between RPM and RPN, we performed pairwise comparisons to obtain differentially expressed genes (DEGs) and identified 1210 genes: 579 genes were upregulated and 631 genes were downregulated (Figure 1; Supplemental Table S2). The top 30 DEGs identified in the RPN and RPM groups are listed in Table 3. As shown in Figure 2, heatmap analysis of the clustering of the identified DEGs showed significant clustering between the two groups. To determine the correlation between samples from different conditions, we performed an OPLS-DA analysis, which showed that the two groups were independent (Figure 3).

### 3.3. Analysis of Differential Gene Enrichment

**Figure 4:**
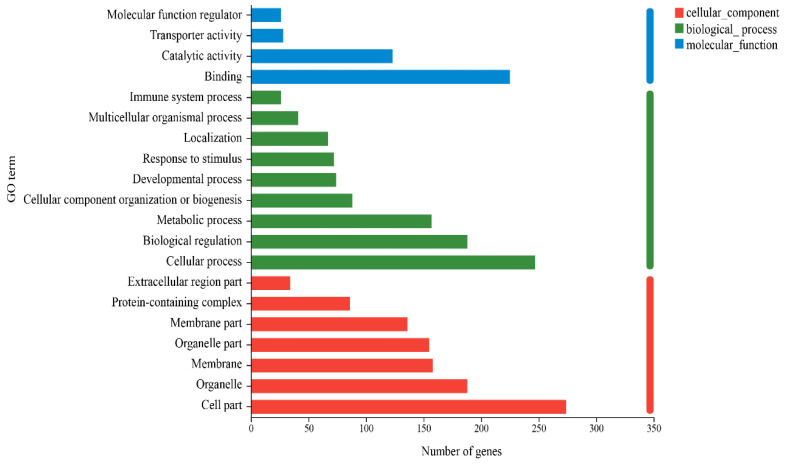
Top 20 GO terms in gene abundance. The ordinate in the graph represents the GO’s secondary classification term, the lower abscissa represents the number of genes/transcripts for that secondary classification on the alignment.

The abundances of the top 20 are shown in Figure 4, and the most prominent GO annotation was for steroid metabolism processes. In these processes, NA and NAM could be involved in the bioprocess of hepatic cholesterol production of steroids and their conversion to other steroids in periparturient dairy cows (Table 4). In addition, the GO results showed that NA and NAM are involved in a variety of molecular reactions in the body by regulating the metabolism of proteins, nucleic acids, and metal ions. For example, NA accelerates cholesterol metabolism, allowing steroid hydroxylase to promote the synthesis of cholesterol derivatives of the steroid hormone cortisol. Cortisol is a common steroid hormone that breaks down triglyceride esters in fats [24]. The KEGG results indicate that monooxygenase is involved in metabolic reactions in the body through various types of electron donors. Monooxygenase requires NAD(P)H to drive the process with O_2_, which is provided by NAM [25].

**Figure 5:**
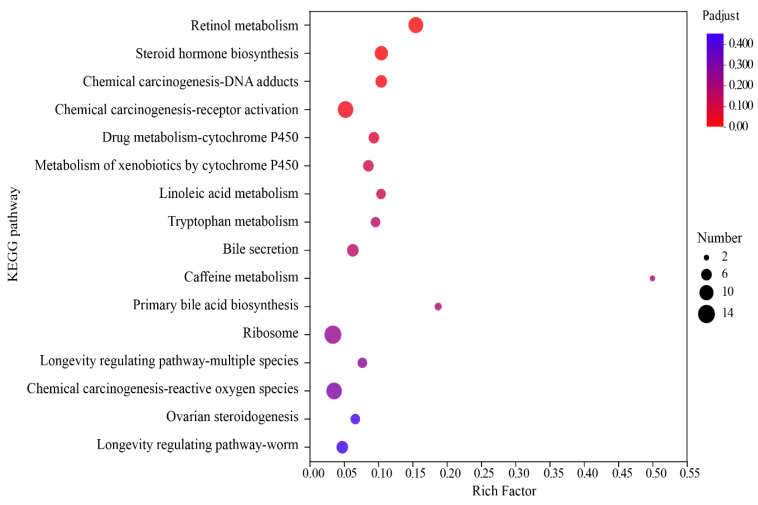
Enriched KEGG pathway of DEGs. The rich factor is the ratio of differentially methylated and expressed gene numbers annotated in this pathway term to all gene numbers annotated in this pathway term. The size of the dot represents the number of genes in the path, and the color of the dot corresponds to different *p*-value ranges.

The results of the pathway enrichment of the DEGs analyzed by KEGG showed that a total of 33 pathways were significantly enriched in two groups and the top 20 enriched KEGG pathways are shown in Table 5. Among these 20 pathways, retinol metabolism had a higher enrichment rate (Figure 5). These results reflected a number of pathway differences between the NA and NAM treatments, which could reveal their different nutritional mechanisms on hepatic metabolism in periparturient dairy cows. 

### 3.4. Validation of DEGs in LO2 Cells

**Figure 6:**
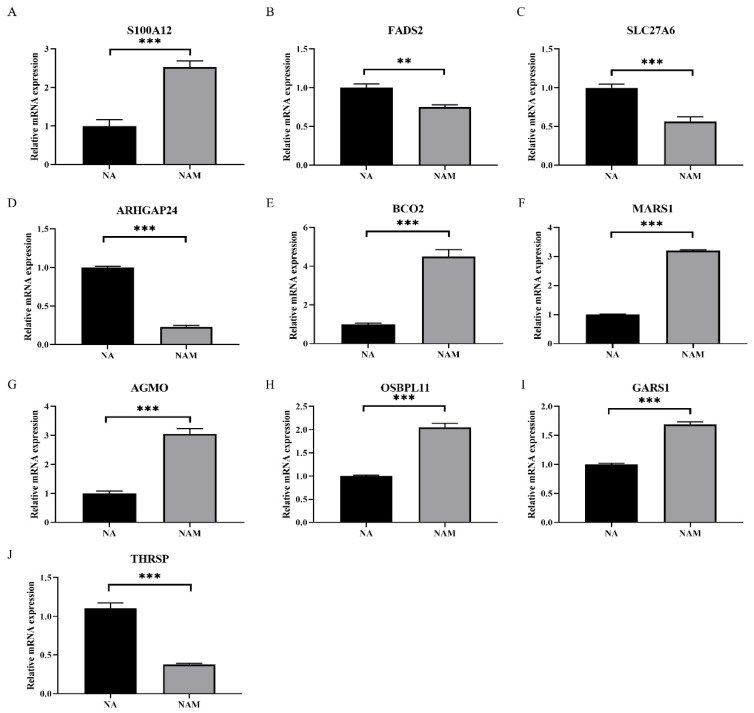
Verification of gene expression in LO2 cells. (**A**) mRNA expression of S100A12. (**B**) mRNA expression of FADS2. (**C**) mRNA expression of SLC27A6. (**D**) mRNA expression of ARHGAP24. (**E**) mRNA expression of BCO2. (**F**) mRNA expression of MARS1. (**G**) mRNA expression of AGMO. (**H**) mRNA expression of OSBPL11. (**I**) mRNA expression of GARS1. (**J**) mRNA expression of THRSP. ** indicates *p* < 0.01, *** indicates *p* < 0.001.

Based on the results of the liver transcriptome analysis, 10 DEGs (*AGMO*, *ARHGAP24*, *BCO2*, *FADS2*, *GARS1*, *MARS1*, *OSBPL11*, *SLC27A6*, *S100A12*, and *THRSP*) in the LO2 cell model were selectively validated by RT-qPCR for the effects of NA and NAM on hepatic nutrient metabolism (Figure 6). The biological relevance, functional diversity, technical feasibility, and significant differential expression observed in the transcriptome of these genes confirmed that these genes play key roles in hepatic metabolic pathways. Compared with the NAM treatment, the NA treatment increased the expression of *FADS2*, *SLC27A6,* and *THRSP* (*p* < 0.05), but reduced the expression of *S100A12*, *ARHGAP24*, *BCO2*, *MARS1*, *AGMO*, *OSBPL11,* and *GARS1* (*p* < 0.05).

## 4. Discussion

The periparturient period of dairy cows is the most critical of their production stages, and the liver, as a central metabolic organ of the whole body, is subject to a variety of nutritional and environmental influences [24]. This study examined the transcriptomic differences in the liver of periparturient dairy cows supplemented with RPN or RPM, and we found most of the DEGs and pathway differences between the two groups were related to lipid metabolism, particularly lipid synthesis. 

Except the differentiated effects on the lipid synthesis pathway, NA and NAM treatments also differed in their involvements in other metabolic pathways, such as steroid synthesis and bile secretion, which reveals that the addition of NA or NAM may indirectly affect lipid metabolism by altering the level of hepatic cholesterol and the metabolism of amino acids, ribosomes, and other important proteins. In order to understand the regulation of nutrients in detail, we will discuss mainly at the genetic level. At the gene level, the top DEG was thyroid hormone responsive spot 14 (*THRSP*). *THRSP* is an important transcription factor in hepatocytes and an adipogenic activator that controls the expression of several genes involved in lipid metabolism, including fatty acid synthase (*FASN*) and stearoyl coenzyme A desaturase 1(*SCD1*) [26]. The levels of glucose, carbohydrates, polyunsaturated fatty acids, and insulin in the liver affect *THRSP* expression [27], the insulin-responsive factor sterol regulatory element-binding protein-1c (*SREBP-1C*) and the glucose-responsive transcription factor, carbohydrate response element binding protein (*ChREBP*), control the levels of *THRSP*. In addition, the upregulation of *THRSP* increases the expression of adenosine triphosphate citrate lyase (*ACL*) and *FASN* in hepatocytes, activates the pregnenolone X receptor (*PXR*), and thus affects steroid metabolism [28]. Cellular assays have shown that NA supplementation can increase glucose and insulin levels compared to NAM and stimulate *THRSP* expression.

In this study, we found the top DEG, *BCO2,* was for retinol metabolism. Vitamin A is stored in hepatic stellate cells in the form of retinol, which provides vitamin A to all tissues in the body [29]. β-carotenoids can be synthesized to vitamin A, which is often described as an antioxidant and is also important for immune responses [30]. Dairy cows are unable to synthesize carotenoids themselves and must obtain them from the diet. Once ingested, they enter the gut where they form micelles with celiac immature chylomicrons and are transferred into the liver [31], where beta-carotene oxygenase 2 (*BCO2*) cleaves them into retinoic acid, the most potent form of vitamin A [32]. However, the excessive accumulation of carotenoids can lead to lipid deposition and a corresponding increase in the risk of oxidative stress and inflammation [31,33]. In this study, the expression of *BCO2* was found to be significantly higher on the NAM treatment than on the NA treatment, suggesting that the addition of NAM may prevent the over-accumulation of hepatic carotenoids and maintain the homeostasis by increasing the expression of *BCO2*.

Periparturient cows are susceptible to hepatic steatosis due to high fat mobilization but inadequate removal of free fatty acids [34]. We further revealed the DEGs that are closely related to liver metabolism to elucidate the differences in regulation of liver metabolism by NA and NAM: *FADS2* and *SLC27A6* were significantly upexpressed on the NA treatment than on the NAM treatment. Fatty acid desaturase 2 (*FADS2*) is a key enzyme in lipid metabolism, and SREBP-1c and PPAR-α may upregulate FADS2 promoter activity [35]. It can convert linolenic acid into long-chain polyunsaturated fatty acids (LC-PUFA), which are essential fatty acids for organisms and play a crucial role in regulating glycolipid metabolism in organisms. [36] In addition, FADS2 may alter the pathogenesis of NAFLD by modifying DNA methylation [37]. The synthesized fatty acids are then transported by specific transport proteins to be used in the body for substance synthesis and other biological processes. Solute carrier family 27 (*SLC27*), consisting of six main members (*SLC27A1*~*SLC27A6*), is commonly referred as fatty acid transporter proteins (FATPs) or very-long-chain acyl-coenzyme A (CoA) synthase (*ACSVLs*) [38]. Studies have shown that *SLC27* can directly take up long-chain fatty acids and also has acyl coenzyme A synthase activity. Acyl coenzyme A catalyzes the conversion of long-chain fatty acids to acyl coenzyme A thioesters for other metabolic processes such as fatty acid oxidation and phospholipid synthesis [39,40]. The differences in the expression of these two genes suggest that NA has a greater ability than NAM to influence hepatic lipid metabolism through regulation of fatty acid synthesis and transport.

Alkyl glycerolipids are used to form a variety of lipids and phospholipids in animals. Alkylglycerol monooxygenase (*AGMO*) is currently the only enzyme capable of degrading alkylglycerol lipids and, due to the irreversible nature of the reaction, is critical for ether lipid metabolism [41]. Ether-linked lipid species act as important biofilm components in several organs of the body and also play key roles in other fatty acid processes, including the degradation of long- and branched-chain fatty acids. Interestingly, *AGMO* is differentially expressed in adipose tissue and liver of hyperlipidemic mice, which implies that its mechanism of action may be different in liver [42,43]. The transcriptome results in this study showed that the *AGMO* expression was significantly higher on the NA treatment than on the NAM treatment, but the cell model confirmed that the *AGMO* expression was higher on the NAM treatment. The exact physiological role of *AGMO* is still unknown. As mentioned above, NA can regulate the glucose and lipid levels of the body, among other things, this may also indirectly affect the condition for *AGMO* activation. The oxysterol binding protein like 11 (*OSBP*)-related protein (*ORP*) family is important in the regulation of cholesterol metabolism and can affect the transport of cholesterol between the Golgi apparatus and biofilm through autophosphorylation, as well as interacting with vitamins after binding [44]. The results of the cell model showed that the expression of *OSBP11* was significantly increased on the NAM treatment, suggesting that NAM may use hydroxysterol binding proteins to influence cholesterol metabolism and thus modulate the status of hepatic steatosis.

As the cow’s pregnancy progresses, coupled with dietary changes, the body goes into a more severe negative energy balance, and oxidative stress and inflammation increase [45]. In addition to fat synthesis and metabolism, NA and NAM supplementation in periparturient dairy cows differed in other aspects, such as oxidative stress and inflammatory factors, in the present study.

Rho GTPase activating protein 24 (*ARHGAP24*) is a member of the ATPase family that can reduce the expression of inflammatory factors, such as IL-6 and TNF-α, by inactivating the *Rac1*/*AKT*/*NF-ĸB* pathway and ameliorate inflammation [46]. The *S100* protein family involves in a variety of cellular processes including apoptosis, migration, protein phosphorylation, differentiation, proliferation, and inflammation [47]. It also binds to pattern recognition receptors, triggering downstream nuclear factor-κB (*NF-κB*), leading to the upregulation of pro-inflammatory gene expression. *S100A12* has several extracellular activities that contribute to the innate immune response, including chemotactic activity and activation of intracellular signaling cascades. It binds to receptors such as *GPCR*, *FGF21*, *TLR4,* and *SR* to produce cytokines that modulate intracellular inflammation and induce oxidative stress [48,49]. It has been shown that *S100A12* is produced by innate immune cells (e.g., granulocytes) and is involved in the chemotaxis of innate immune cells. It can exist as a dimer or oligomer and can bind divalent cations, including zinc, copper, and calcium, to promote “nutritional immunity” to invading microbial pathogens. *S100A12* interacts with cell surface membranes as well as *RAGE*, *TLR4,* and *CD36* receptors to promote pro-inflammatory signaling and disease progression and is a potential biomarker for the early diagnosis of inflammation [50]. In this study, we found that the expression of S100A12 was significantly lower on the NA treatment than on the NAM treatment; the results for *ARHGAP24* were opposite, suggesting that the addition of NA reduces inflammation and apoptosis by affecting downstream signaling by decreasing the expression of *S100A12* and increasing the expression of *ARHGAP24* in the liver of cows.

Methionyl-tRNA synthetase 1 (*MARS1*) and glycyl-tRNA synthetase 1 (*GARS1*) both belong to the group of aminoacyl-tRNA synthases (*ARSs*). ARSs are generally used for protein synthesis and interact with proteins in the mTORC1, GCN2, CDK4, and vascular endothelial growth factor receptor (*VEGFR*) signaling pathways and are potential markers of pneumonia. Several studies have found that inhibitors of ARSs have been used in therapeutic trials for a variety of diseases [51,52,53]. In this study, *MARS1* and *GARS1* were found to be significantly downregulated on the NA treatment, suggesting that NA modulates inflammation and other intracellular pathways via tRNA synthase, thereby improving liver metabolism.

In the present study, we also found that the major differential pathways between NA and NAM focused on the processes of steroid catabolism, steroid hydroxylase, monooxygenase activity, oxidoreductase activity, hemoglobin binding, and ferric iron binding, mostly lipid anabolism and redox processes, suggesting that NA was more effective than NAM in regulating these metabolic processes. Due to the fact that NA has its own specific receptor, *GPR109A*, it directly or indirectly increases hepatic lipid oxidation and reduces the ab initio synthesis of fatty acids, and also non-competitively inhibits the activity of key rate-limiting triglyceride enzymes in the liver to alleviate hepatic lipid deposition. NAM acts more indirectly as a precursor of NAD and NADP, which in turn increases the hepatic expression of the *SIRTI*, *SIRT2*, *SIRT3,* and *SIRT6* genes through activation of the NAD-sirtuin pathway. NAM is more efficient than NA under the same conditions [54], maintains the levels of reduced glutathione and thioredoxin in the antioxidant system, and is involved in processes such as cellular energy metabolism [45].

## 5. Conclusions

In this study, we investigated the effects of RPN and RPM on the liver transcriptome of periparturient dairy cows, identified 1210 DEGs and 33 different KEGG pathways, and 10 significantly differential genes were validated in LO2 cells. Although both NA and NAM can reduce hepatic lipid deposition, they have different mechanisms of trophic action. Compared with the niacinamide group, the NA group could upregulate the expression levels of *FADS2*, *SLC27A6*, *ARHGAP24*, and *THRSP*, and downregulate the inflammatory oxidative stress-related genes *S100A1*, *2 MARS1*, *GARS1*, etc. The addition of NA acid may affect glucose and insulin levels and thus the expression of some genes; in addition, NAM can upregulate the expression levels of *BCO2* and *OSBP11*. In summary, compared with RPM, RPN generally directly regulated fatty acid anabolism and transport, inflammatory factor expression and oxidative stress processes, and played a prominent role in liver metabolism. Whereas, RPM acted more as a precursor of NAD and NADP to indirectly participate in biological processes such as ether lipid metabolism, cholesterol metabolism, energy metabolism, and other processes.

In addition, the cell results showed an optimum addition dosage of NAM at a higher level than NA (NAM:NA = 2:1), which means that the cost of adding NAM to dairy cow diets may be higher, but if cost is ignored, NAM is better and more directly effective. Perhaps in the future we will combine the two nutrients to achieve a ratio that is both cost effective and the most beneficial to the nutritional health of the animal.

## Figures and Tables

**Table 1:** Ingredient composition of the diets fed during the prepartum and postpartum periods.

	Prepartum	Postpartum
Ingredients, DM%		
Corn silage	22.67	30.36
Oat hay	8.12	4.55
Straw	39.35	0.00
Alfalfa hay	0.00	14.43
Flaking corn	0.00	15.92
Soybean meal	0.00	3.06
Cottonseed meal	0.00	4.54
NaHCO_3_	0.00	0.95
1512 ^1^	29.86	0.00
1818 ^2^	0.00	16.10
1979 ^3^	0.00	10.08
^4^ Chemical composition, DM%		
NE_L_ (Mcal/kg)	1.30	1.69
CP	10.97	17.49
EE	3.48	4.22
Ash	8.11	8.71
NDF	52.10	36.39
ADF	28.34	20.53
Starch	14.00	25.00
Ca	0.87	0.93
*p*	0.56	0.57

^1^ A total of 1512 concentrate supplements are available to the TMR per kg (dry matter base): 286.35 mg Fe, 33.64 mg Cu, 205.65 mg Zn, 162.57 mg Mn, 1.06 mg Se, 102 mg Co, 1.83 mg I, 19.27 IU vitamin A, 6.64 IU vitamin D, and 82.14 IU vitamin E. ^2^ A total of 1818 concentrate supplements are available to the TMR per kg (dry matter base): 349.23 mg Fe, 61.42 mg Cu, 319.01 mg Zn, 278.59 mg Mn, 1.83 mg Se, 201 mg Co, 3.57 mg I, 27.87 IU vitamin A, 9.89 IU vitamin D, and 95.73 IU vitamin E. ^3^ A total of 1979 concentrate supplements are available to the TMR per kg (dry material base): 141.04 mg Fe, 67.58 mg Cu, 304.56 mg Zn, 284.77 mg Mn, 1.79 mg Se, 100.6 mg Co, 3.57 mg I, 27.01 IU vitamin A, 9.92 IU vitamin D, and 183.35 IU vitamin E. ^4^ All values are measured, except for net lactation energy and starch values, which are calculated values.

**Table 2:** Sequences of primers for PCR.

Target Gene	Forward (5′-3′)	Forward (5′-3′)
*S100A12*	GCTCAGTGCCCTTCACCACT	AGCCTTCAGCGCAATGGCTA
*FADS2*	ACCGTGACTGGTTCAGTAGC	CGGGGCGATCTTGTGTAAGT
*SLC27A6*	GAAGAGAAGGACGCTGGTGG	AAGTGCAGGACGACCATTCC
*ARHGAP24*	GTATCGCCAATGCAGGATGC	CGTGGGCATGGTTTAGCAAG
*BCO2*	GGCTGATGGAACGATCTGGT	CCCCACTAAATGCCGAAAGC
*MARS1*	TCCATGCTGACATCTACCGC	CAGTGCTCACATCGCAGTTG
*AGMO*	ACTACATTCTGGGCCACACC	GGGAACTTCTTTGCCGGTGA
*OSBPL11*	ATGAAGCTGGGCTGTTGGAG	GTGCTGTCGCTCTTTTGCAT
*GARS1*	TCTCCCCTCGATCTGGACTG	TGACCTGGGCTTTTGCTGAA
*GAPDH*	AAGGTCGGAGTGAACGGATTC	ATTGATGGCGACGATGTCCA
*THRSP*	TGAGGCCCCTGATCTCTACA	CTTCCTCTGTCTCTGCGGTT

The 2^−∆∆Ct^ method was used to determine the relatively quantitative gene expression levels, normalized by GAPDH.

**Table 3:** The top 30 DEGs identified in the RPN and RPM treatments.

Gene Name	Gene Description	Log_2_FC (RPN/RPM)	*p*-Value
*THRSP*	Thyroid hormone responsive	5.7793	7.50 × 10^−17^
*CYP26A1*	Cytochrome P450, family 26, subfamily A, Polypeptide 1	3.6386	2.23 × 10^−16^
*MT1E*	Metallothionein 1E	−2.6549	1.84 × 10^−12^
*ARMH1*	Armadillo like helical domain containing 1	−2.1066	1.98 × 10^−11^
*CYP2C19*	Cytochrome P450, family 2, subfamily C, Polypeptide 19	2.6907	3.39 × 10^−11^
*SEC14L3*	SEC14 like lipid binding 3	4.0599	1.46 × 10^−10^
*CES1*	Carboxylesterase 1 (monocyte/macrophage serine esterase 1)	1.4039	3.15 × 10^−10^
*KCNN2*	Potassium calcium-activated channel Subfamily N member 2	2.2039	3.52 × 10^−10^
*S100A12*	S100 calcium binding protein A12	−3.2975	9.00 × 10^−10^
*ATP6V1C2*	ATPase H^+^ transporting V1 subunit C2	−3.7203	9.22 × 10^−9^
*PLXNB1*	plexin B1	−1.5450	1.12 × 10^−8^
*FADS2*	Fatty acid desaturase 2	2.7283	1.18 × 10^−8^
*SLC27A6*	Solute carrier family 27 member 6	1.6232	1.86 × 10^−8^
*HSD17B6*	Hydroxysteroid (17-beta) dehydrogenase 6	1.1792	2.40 × 10^−8^
*ARHGAP24*	Rho gtpase activating protein 24	1.1679	6.69 × 10^−8^
*MUSTN1*	Musculoskeletal, embryonic nuclear protein 1	−2.2457	1.30 × 10^−7^
*BCO2*	Beta-carotene oxygenase 2	1.2256	1.45 × 10^−7^
*ANO1*	Anoctamin 1	1.3874	1.72 × 10^−7^
*TRPM2*	Transient receptor potential cation channel subfamily M member 2	1.6755	1.87 × 10^−7^
*NT5DC2*	5’-nucleotidase domain containing 2	−1.2762	2.71 × 10^−7^
*ECD*	Ecdysoneless cell cycle regulator	−1.3113	3.05 × 10^−7^
*MARS1*	Methionyl-trna synthetase 1	−2.0929	6.92 × 10^−7^
*BACE1*	Beta-secretase 1	−1.3440	7.30 × 10^−7^
*AGMO*	Alkylglycerol monooxygenase	1.3366	8.96 × 10^−7^
*ODF2L*	Outer dense fiber of sperm tails 2 like	1.6741	1.00 × 10^−6^
*OSBPL11*	Oxysterol binding protein like 11	2.2486	1.08 × 10^−6^
*WFS1*	Wolframin ER transmembrane glycoprotein	−1.6581	1.24 × 10^−6^
*JARID2*	Jumonji and AT-rich interaction domain containing 2	1.9052	1.31 × 10^−6^
*GARS1*	Glycyl-trna synthetase 1	−1.0204	1.79 × 10^−6^
*SPOCK1*	SPARC (osteonectin), cwcv and kazal like domains proteoglycan 1	2.4819	1.97 × 10^−6^

RPN = rumen-protected niacin; RPM = rumen-protected nicotinamide.

**Table 4:** The top 20 enriched GO terms among DEGs.

GO ID	Category	Description	*p*-Value	Counts
GO:0006706	BP	Steroid catabolic process	1.29 × 10^−7^	6
GO:0006805	BP	Xenobiotic metabolic process	3.08 × 10^−7^	11
GO:0008395	MF	Steroid hydroxylase activity	1.43 × 10^−7^	10
GO:0004497	MF	Monooxygenase activity	1.80 × 10^−7^	15
GO:0020037	MF	Heme binding	2.34 × 10^−7^	16
GO:0005506	MF	Iron ion binding	3.34 × 10^−7^	17
GO:0046906	MF	Tetrapyrrole binding	4.29 × 10^−7^	16
GO:0016705	MF	Oxidoreductase activity, acting on paired Donors, with incorporation or reduction in molecular oxygen	4.77 × 10^−7^	18
GO:0008202	BP	Steroid metabolic process	8.64 × 10^−7^	13
GO:0120254	BP	Olefinic compound metabolic process	1.16 × 10^−6^	10
GO:0016491	MF	Oxidoreductase activity	2.03 × 10^−6^	36
GO:0044281	BP	Small molecule metabolic process	4.08 × 10^−6^	40
GO:0044237	BP	Cellular metabolic process	5.54 × 10^−6^	145
GO:0001523	BP	Retinoid metabolic process	5.76 × 10^−6^	7
GO:0016101	BP	Diterpenoid metabolic process	7.35 × 10^−6^	7
GO:0070988	BP	Demethylation	8.27 × 10^−6^	7
GO:0008152	BP	Metabolic process	1.03 × 10^−5^	157
GO:0046872	MF	Metal ion binding	1.02 × 10^−5^	87
GO:0016712	MF	Oxidoreductase activity, acting on paired donors, with incorporation or reduction in Molecular oxygen, reduced flavin or flavoprotein as one donor, and incorporation of one atom of oxygen	1.03 × 10^−5^	6
GO:0006721	BP	Terpenoid metabolic process	1.77 × 10^−5^	7

BP = biological process; CC = cellular component; MF = molecular function.

**Table 5:** The top 20 enriched KEGG pathways of DEGs.

Pathway ID	KEGG Pathway	*p*-Value	Counts
map00830	Retinol metabolism	3.12 × 10^−9^	11
map00140	Steroid hormone biosynthesis	2.66 × 10^−6^	9
map05204	Chemical carcinogenesis-DNA adducts	3.54 × 10^−5^	7
map05207	Chemical carcinogenesis-receptor activation	7.76 × 10^−5^	12
map00982	Drug metabolism-cytochrome P450	0.00024	6
map00980	Metabolism of xenobiotics by cytochrome P450	0.00039	6
map00591	Linoleic acid metabolism	0.00048	5
map00380	Tryptophan metabolism	0.00070	5
map04976	Bile secretion	0.00083	7
map00232	Caffeine metabolism	0.00109	2
map00120	Primary bile acid biosynthesis	0.00124	3
map03010	Ribosome	0.00168	14
map04213	Longevity regulating pathway-multiple species	0.00194	5
map05208	Chemical carcinogenesis-reactive oxygen species	0.00236	12
map04913	Ovarian steroidogenesis	0.00363	5
map04212	Longevity regulating pathway-worm	0.00412	7
map00983	Drug metabolism-other enzymes	0.00587	5
map01040	Biosynthesis of unsaturated fatty acids	0.00932	3
map04923	Regulation of lipolysis in adipocytes	0.00964	4
map04978	Mineral absorption	0.01155	5

## Data Availability

All data relevant to the study are included in the article or uploaded as supplementary information.

## Supplementary Materials

The following supporting information can be downloaded at: https://www.mdpi.com/article/10.3390/metabo14030150/s1, Supplementary Figure S1: Venn analysis between RPN and RPM group; Supplementary Table S1: Statistics for quality control of sequencing data; Supplementary Table S2: Differential gene statistics of up and down regulation.
