# Supplementary figures and images for: Transcriptome in Liver of Periparturient Dairy Cows Differs between Supplementation of Rumen-Protected Niacin and Rumen-Protected Nicotinamide
Source: Metabolites. 2024 Mar 1;14(3):150. doi: 10.3390/metabo14030150 (PMC10972113; doi:10.3390/metabo14030150)

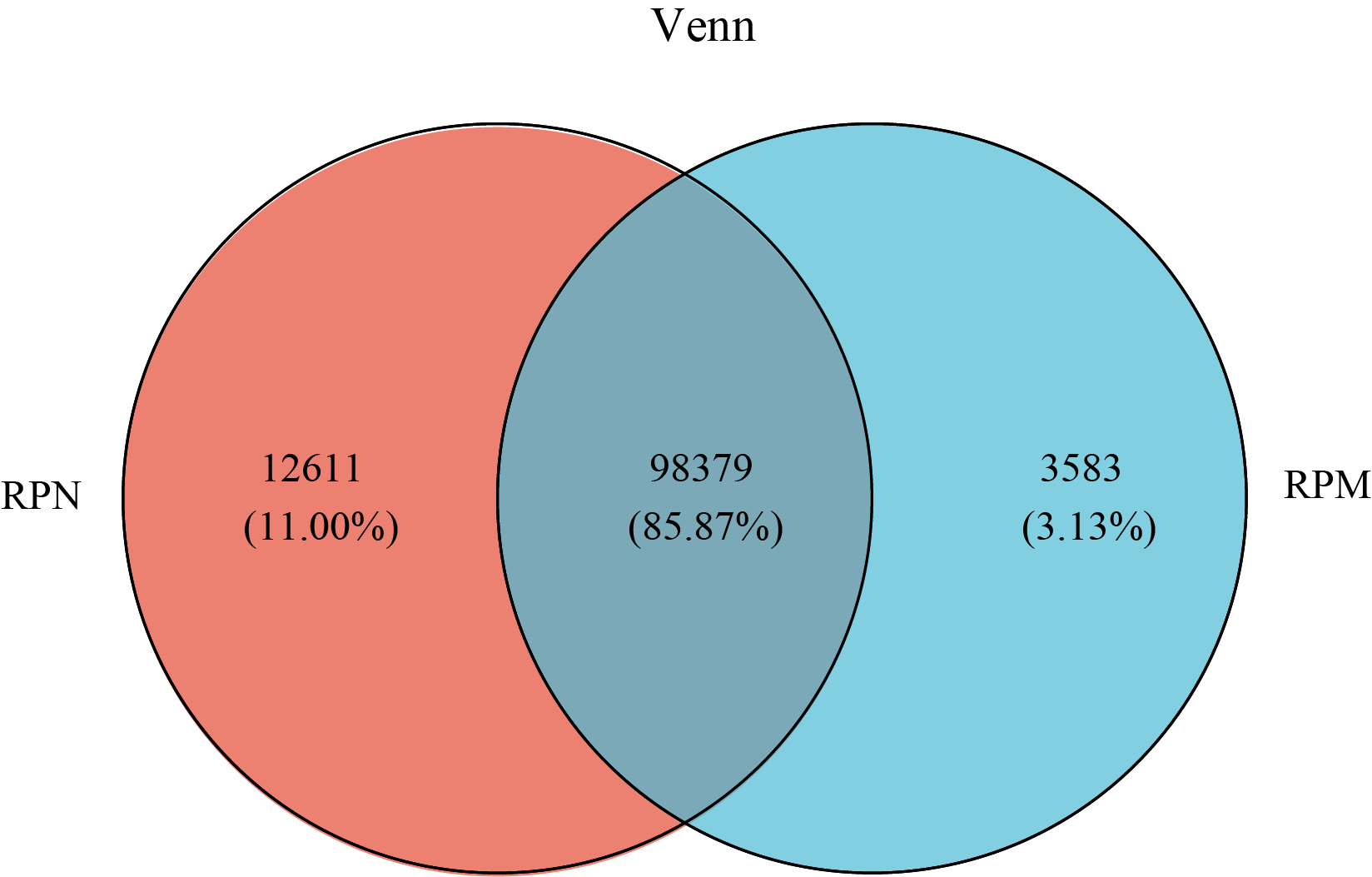

Supplement: Supplementary file 1 [file metabolites-14-00150-s001.zip › supporting information/Supplementary Figure S1.png]
